# Solutions to the Drawbacks of Photothermal and Photodynamic Cancer Therapy

**DOI:** 10.1002/advs.202002504

**Published:** 2021-01-05

**Authors:** Xiangyu Deng, Zengwu Shao, Yanli Zhao

**Affiliations:** ^1^ Department of Orthopaedic Surgery Union Hospital Tongji Medical College Huazhong University of Science and Technology Wuhan 430022 China; ^2^ Division of Chemistry and Biological Chemistry School of Physical and Mathematical Sciences Nanyang Technological University 21 Nanyang Link Singapore 637371 Singapore

**Keywords:** cancer treatment, clinical potential, photodynamic therapy, phototherapy, photothermal therapy

## Abstract

Phototherapy such as photothermal therapy and photodynamic therapy in cancer treatment has been developed quickly over the past few years for its noninvasive nature and high efficiency. However, there are still many drawbacks in phototherapy that prevent it from clinical applications. Thus, scientists have designed different systems to overcome the issues associated with phototherapy, including enhancing the targeting ability of phototherapy, low‐temperature photothermal therapy, replacing near‐infrared light with other excitation sources, and so on. This article discusses the problems and shortcomings encountered in the development of phototherapy and highlights possible solutions to address them so that phototherapy may become a useful cancer treatment approach in clinical practice. This article aims to give a brief summary about current research advancements in phototherapy research and provides a quick guideline toward future developments in the field.

## Graphical abstract

This article discusses the issues and shortcomings encountered in the development of photodynamic and photothermal therapy and highlights possible solutions to address them so that phototherapy may become a useful cancer treatment approach in clinical practice.

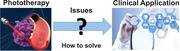

## 1 Introduction

According to the global burden of disease published in the 2017 Lancet article, tumors are still the chronic and non‐communicable diseases with high mortality and deaths in the world.^[^ ^1^ ^]^ The treatment of tumors has always been an urgent need to be addressed in various countries. In addition to the continuous improvement and innovation of traditional treatments such as surgical resection, radiotherapy, and chemotherapy,^[^ ^2^, ^3^, ^4^ ^]^ many new treatments have gradually entered the stage. For example, tumor immunotherapy has gained a great progress, especially in the treatment of hematological tumors.^[^ ^5^, ^6^, ^7^ ^]^ Rapid development of medical‐industrial integration also brings new ideas to tumor treatment. Biomaterials for tumor treatment are one of the most widely developed and applied interdisciplinary examples.^[^ ^8^, ^9^, ^10^ ^]^ Among them, photosensitive materials account for an important part. Photosensitive materials mainly include photothermal materials and photodynamic materials. Nanomaterials that convert light to heat are capable of absorbing certain kinds of light, especially near‐infrared light (NIR). These nanomaterials with wide light wavelength range possess unique optical properties, enabling the skylight to the lesion site through the skin and tissue of the human body.

Phototherapy approach often requires three main components, including light irradiation (i.e., NIR light), photosensitizers and oxygen (O_2_). NIR light is the trigger point and also the energy source. NIR light may also reduce changes to the features and conformation of amino acids, and it can stimulate aggregation to specific proteins. The development of different and adjustable NIR wavelengths would expand the therapeutic field. Photosensitizers act as intermediary agents, which can transfer the energy of NIR into other forms to be used in cancer cell killing process. There are many kinds of photosensitizers. In addition to small molecule dyes, supramolecular complexes and conjugated polymers, some natural photosensitizers like curcumin and chlorophyll derivatives have also been found to have light‐responsive characteristics. The development of new photosensitizers may accelerate the practical applications of phototherapy approach. Oxygen is the substrate to produce toxic reactive oxygen species (ROS) in photodynamic therapy (PDT). Adequate oxygen supply is the prerequisite to ensure a sufficient effect of PDT. As tumor hypoxia microenvironment could promote the tumor growth and metastasis, reversing tumor hypoxia is an important strategy in the tumor treatment.

Photothermal therapy (PTT) uses the thermal energy induced by light‐to‐heat conversion materials to kill cancer cells. The heat generated by plasmon resonance or energy transition zone causes local high temperature without affecting the remaining normal tissues, and ultimately kills the tumor cells that endocytose the photothermal materials. Because of unique characteristics, functional materials with photothermal conversion properties are favored in biological applications, and many biomaterials have been developed and applied to the PTT‐based cancer treatment filed.^[^ ^11^, ^12^, ^13^, ^14^, ^15^, ^16^ ^]^

PDT is another method of treating tumor disease with photosensitizing drugs and light activation. Irradiating the tumor site with a specific wavelength can activate the photosensitizing drugs that are selectively concentrated in tumor tissues, triggering a photochemical reaction to destroy the tumor cells. Photosensitizing drugs transfer energy to the surrounding oxygen, producing highly active singlet oxygen (^1^O_2_). Singlet oxygen can oxidize nearby biological macromolecules, produce cytotoxicity and kill tumor cells.^[^ ^17^, ^18^, ^19^, ^20^, ^21^ ^]^ Compared with traditional treatment methods, PDT has the advantage to perform effective treatment accurately with minimal side effects. More importantly, many studies have shown that PDT can induce immune cell death (ICD) in tumor cells.^[^ ^22^, ^23^, ^24^ ^]^ ICD is an apoptotic form characterized by the release of multiple damage‐related molecular signals, which provides a new theoretical guidance in current immunotherapy.^[^ ^25^, ^26^, ^27^ ^]^ Through immunogenic death, many cancers with poor immunogenicity can be reversed, because the explosion of antigens can enhance the reaction of immune cells. One of the most important signs of immunogenic death is the translocation of calreticulin from the endoplasmic reticulum to the outside of the cell membrane, promoting the phagocytosis and presentation of tumor antigens by dendritic cells, thereby effectively initiating specific antitumor immune responses.^[^ ^28^, ^29^, ^30^ ^]^ Due to the important role of immunogenic death in cancer immunotherapy, the development of drugs that induce immunogenic death of cancer cells has been receiving much attention.^[^ ^29^, ^31^, ^32^, ^33^ ^]^ However, effective immunogenic death inducers are currently very limited. In addition to a few chemotherapeutic drugs and photosensitizers, oncolytic viruses, gamma rays, and ultraviolet radiation have been reported to cause immunogenic death of cancer cells.^[^ ^30^ ^]^ Photosensitizers not only achieve the effect of PTT and PDT, but also induce immunogenic death, a process that promotes the body's active antitumor immunity.

**Scheme 1:**
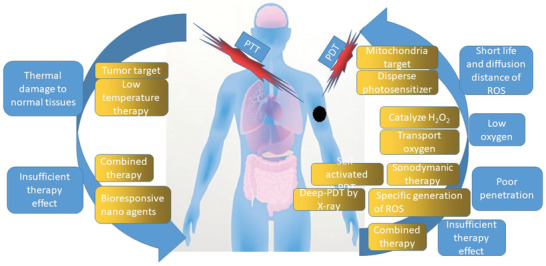
Schematic presentation of phototherapy. Drawn by Adobe Illustrator.

While phototherapy in the field of antitumor research has been developing rapidly, most of the approaches still cannot be applied into clinical transformation. The reason is closely related to defects of photosensitive materials in the cancer treatment. For example, the thermal damage of normal tissues caused by PTT, and the short life span and short diffusion distance limit PDT, which weaken their antitumor performance. Therefore, this article discusses the problems and shortcomings encountered in the development of phototherapy and highlights possible solutions. The article starts with the limitations of phototherapy, summarizes the solutions proposed by researchers in recent years aiming to solve the problems of phototherapy, and provides more comprehensive understanding of phototherapy in antitumor research. The article content includes solutions for thermal damage to normal tissues caused by PTT,^[^ ^34^, ^35^, ^36^, ^37^, ^38^, ^39^, ^40^, ^41^ ^]^ solutions for insufficient treatment effect of PTT, solutions for extremely short life and short diffusion distance of ROS in PDT, solutions for lack of oxygen that can produce ROS in PDT, solutions for poor penetration effect of excitation light source, and solutions for insufficient treatment effect of single PDT. **Scheme** **1** is the sketch map of this topic. There are two main phototherapy methods, i.e., PTT and PDT. Each of them has drawbacks with possible solutions developed by scientists. We have listed as many of these solutions as possible in order to provide a quick guideline in the phototherapy of cancer.

## 2 Solutions for the Defects in Photothermal Therapy

### 2.1 Solutions for Thermal Damage to Normal Tissues Caused by Photothermal Therapy

The efficient photothermal treatment is based on the high target ability of photosensitizers to tumor site. Only high concentration in the tumor site and the absence of photosensitizer localization in normal issues exert the largest agglomerative thermal effect, so as to achieve a fixed‐point temperature rise and then “heat” the tumor, while the normal tissues would not be damaged by the heat. Researchers have proposed two solutions that guarantee the tumor‐killing effect and ensure the safety of normal tissues. One is to improve the targeting ability of photosensitizers, and the other is to apply low‐temperature PTT.

When improving the targeting ability, the photosensitizing materials should be highly concentrated in the tumor site. Under the excitation of NIR light, only the tumor site where the photosensitizers are present would generate large amount of thermal energy, which does not occur in normal tissues. While low temperature photothermal treatment is to reduce the heat resistance of tumor cells by inhibiting the synthesis of heat shock proteins (HSPs), improve the effect of photothermal treatment to tumors, and reduce the temperature required for the treatment. This concept was developed by Liu and coworkers,^[^ ^39^ ^]^ and then many scientific studies strengthened and optimized this method to obtain better tumor suppression effect and maximally reduce thermal damage to normal tissues.

#### 2.1.1 Improving Targeting Ability of Photothermal Materials

If tumor‐specific targeting agents are not added, the tumor‐targeting capabilities of most photothermal materials would depend on enhanced permeability and retention (EPR) effect, which means macromolecules and particles with specific sizes (such as liposomes and vesicles) are more likely to penetrate into tumor tissues by the EPR effect and remain there for a certain period of time (compared to normal tissues). Researchers believe that tumor cells need more nutrients and oxygen to maintain their quick growth rate, so they secrete vascular endothelial growth factor. Especially when the tumor reaches 150–200 µm in size, it would be highly dependent on the nutrition and oxygen supply that tumor blood vessels provide. At this time, the newly generated tumor blood vessels are very different in structure and morphology from normal blood vessels. The endothelial cell gap is large, the smooth muscle layer of the blood vessel wall is missing, and the function of angiotensin receptor is absent. In addition, the lack of lymphatic vessels in tumor tissues prevents the return of lymph fluid. These factors enable macromolecular substances to easily pass through the blood vessel wall and enrich in tumor tissues, and these substances cannot be carried away by the return of lymph fluid so they would retain in the tumor tissues for a long period of time. If the size, properties, surface characteristics, porosity, composition, and targeting ligands of the photothermal materials are optimized, the EPR effect would maximize the targeting effect, giving rise to the maximum tumor lethality.

Du and coworkers^[^ ^42^ ^]^ designed a collective nanoplatform named as iCluster, which can reduce it size from about 100 nm to about 5 nm at the tumor site. The feature significantly improves the perfusion of particles in the primary tumor stroma, revealing that this tumor‐specific size transition promotes the aggregation of particles in the tumor tissue and also in lymphatic vessels. This is a typical approach to enhance the targeting ability of nanoparticles by changing the size to improve the efficiency of PTT. Another method to enhance specific targeting ability is to load tumor targeting ligands on nanoparticles, so that the nanoparticles can tightly bind to tumor cells. The most widely used targeting ligands include folic acid^[^ ^43^, ^44^, ^45^, ^46^, ^47^ ^]^ and hyaluronic acid,^[^ ^48^, ^49^, ^50^ ^]^ since their receptors are over‐expressed in most solid tumors due to rapid growth characteristics of malignant tumors. Some special receptors only express on the surface of certain types of tumor cells. For example, Qian and coworkers^[^ ^51^ ^]^ used the specific receptor of breast cancer to design nanomaterials in order to enhance the targeting effect. As p32 protein is over‐expressed on breast cancer cells (surface and intracellular) as well as tumor‐associated lymphocytes and tissues, the authors modified the Lyp‐1 ligand on the nanoparticle surface to specifically target p32 protein. Specific recognition and uptake of nanoparticles increase the antitumor effect. The in vitro and in vivo results show that Lyp‐1 modification enhances the ability of targeted delivery to tumors and effectively inhibits the growth and metastasis of mouse breast cancer by combining PTT/PDT/chemotherapy. Thus, by controlling the size and characteristics of nanoparticles, and increasing the surface ligands of nanoparticles, the targeting effect of biomaterials to tumors could be improved, thereby solving the side effects of thermal damage to normal tissues caused by PTT. The targeting ability can be gained by these methods. With more ligands and proteins loaded in nanoparticles, there may be less drugs and less active ingredients in the system, so that the antitumor effect may be weakened. Thus, the balance of targeting ability and lethal efficacy should be taken into consideration in the materials design process.

#### 2.1.2 Low‐Temperature Photothermal Therapy

As discussed above, the low‐temperature PTT is proposed to solve the problem of thermal damage to normal tissues due to thermal diffusion during the therapy process. However, how to ensure the tumor killing effect even at relatively low temperature (43–45 °C) is the most concerned issue in this specific research field.

**Figure 1:**
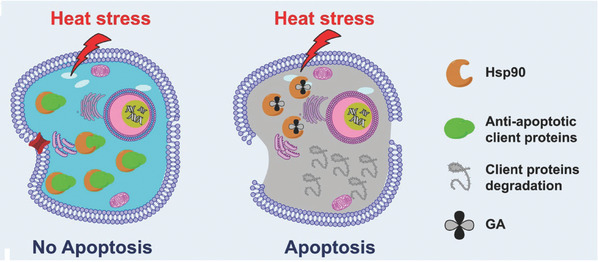
Illustration showing the use of GA‐containing nanosystem to overcome thermal resistance by inhibiting HSP90. Reproduced with permission.^[39]^ Copyright 2017, WILEY‐VCH Verlag GmbH & Co.

Liu and coworkers^[^ ^39^ ^]^ proposed that low‐temperature PTT is based on the inhibition of HSPs, which are a class of heat emergency proteins widely present in bacteria and mammals. This protein is synthesized to protect itself when the organism is exposed to high temperature. Therefore, inhibiting the synthesis of HSPs can reduce the heat resistance ability of tumor cells, thereby enhancing the effect of tumor PTT and reducing the temperature required for the treatment. **Figure** **1** shows the mechanism of HSP inhibition. In this research, gambogic acid (GA) acts as the simulant of antiapoptotic client proteins. When GA exists, apoptosis would be activated even the temperature is relatively low. The authors constructed PEGylated 1D metal‐organic framework (MOF) by the phase transfer method. This method is simple and applicable to different types of metal ions. The 1D MOF not only has good dispersibility and long blood circulation time, but also realizes charge reversal in acidic tumor microenvironment, thereby directly improving the retention and enrichment of the drug carrier in the tumor site. Upon loading the HSP inhibitor (i.e., GA), the nanosystem destroys the tumor at relatively low temperature (below 45 °C) by inhibiting the expression of HSP90. The nanosystem achieves low temperature heating‐induced effective cancer cell apoptosis. It is worth mentioning that this 1D MOF is biodegradable and can be excreted by the kidney, so that the long‐term toxicity of the nanosystem can be alleviated, which is helpful for future clinical trials.

Similarly, Shao and coworkers^[^ ^35^ ^]^ loaded SNX‐2112, a small molecule inhibitor of HSP90, on 2D graphene nanoplatform to achieve the effect of low‐temperature PTT. They found that, during this process, low‐temperature PTT triggered excessive autophagic death of tumor cells, and this therapy method can achieve a long‐term tumor suppression effect through the activation of body's immune system. In order to realize the effect of low‐temperature PTT, Wu and coworkers^[^ ^52^ ^]^ designed a multifunctional temperature‐sensitive liposome. The temperature‐sensitive phase change liposome was used to encapsulate glucose oxidase (GOx), indocyanine green (ICG) and GA. The drug‐loaded temperature‐sensitive liposome achieved thermal responsive delivery of multiple drugs in the tumor site along with low temperature hyperthermia. Both cell and animal experiments showed that this tumor starvation and phototherapy strategy significantly improved the therapeutic efficiency by mild PTT. This strategy provides a feasible and reliable method for solving the contradiction between low‐temperature photothermal efficiency and dosage. More importantly, by using enzyme‐mediated tumor starvation and phototherapy to enhance the efficiency of low‐temperature PTT, this strategy is expected to accelerate the clinical transformation of the PTT approach.

Recently, Chen and coworkers^[^ ^53^ ^]^ prepared a scaffold material that can respond to outside stimulation. The polymer scaffold was able to respond pH‐triggered mild PTT. The system can induce tumor cell apoptosis and significantly improve the growth of adipose‐derived stem cells. Experiments show that low‐temperature photothermal treatment using this scaffold material can induce more than 95% of the cell death in human breast cancer cells (MCF‐7) in vitro, as well as completely inhibit the tumor growth in vivo and eventually eliminate tumor tissue in mice. At the same time, the polymer scaffold can increase the ability of adipose‐derived stem cells to differentiate into adipocytes by up‐regulating the expression of fat‐related genes. No matter with or without NIR light irradiation, it can significantly promote the formation of new adipose tissue. These results indicate that the polymer scaffold with bifunctional properties has promising clinical application prospects.

All the above examples are the solutions proposed by researchers in order to achieve a strong tumor suppression effect, while protecting normal tissues from thermal damage as much as possible. Many of them obtained some good results, presenting the value of clinical translation in the future.

Low‐temperature photothermal effect is still a controversial treatment method, for its relatively short observation time and insufficient research data. Although HSP inhibitor and other drugs can be introduced into the system, the antitumor effect and possible side effect still require more comprehensive investigations. Nevertheless, it is an innovative medical‐integrative treatment and shows a great potential for future clinical application.

### 2.2 Solutions for Insufficient Photothermal Effect

The biggest problem of PTT is the limited penetration depth of light, which leads to incomplete cure of tumors outside the radiation range. Monotherapy is usually not enough to completely cure the tumor, and PTT is not an exception. Even with high therapeutic effect by PTT, its own limitations may still result in incomplete elimination of cancer cells, which in turn lead to the tumor recurrence and metastasis. The combination of PTT with other therapeutic approaches would improve the overall treatment efficacy. In many cases, the combination of different therapeutic approaches does not just provide a simple supplement, but a synergistic treatment effect.^[^ ^54^ ^]^

#### 2.2.1 Combined Therapy

A common strategy is the combination of PTT with chemotherapy.^[^ ^55^, ^56^, ^57^, ^58^, ^59^ ^]^ For instance, Zhang and coworkers^[^ ^60^ ^]^ succeeded in functionalizing molybdenum telluride nanosheets (MoTe_2_‐PEG‐cRGD) followed by loading with chemotherapeutic doxorubicin (DOX) drug for tumor diagnosis and treatment. The obtained MoTe_2_‐PEG‐cRGD/DOX has high light‐to‐heat conversion efficiency and exhibits good cell killing ability under NIR irradiation. Due to the specific tumor targeting mediated by the cyclic arginine‐glycine‐aspartate (cRGD) motif, MoTe_2_‐PEG‐cRGD/DOX shows efficient accumulation in the tumor and strong tumor cauterization effect. Importantly, MoTe_2_‐PEG‐cRGD can degrade under the stimulation of NIR light. The in vitro and in vivo experiments indicate that this nanosystem has tumor targeting function and high photothermal conversion efficiency, successfully realizing the tumor diagnosis and combined photothermal/chemotherapy treatment. In another example, Lam and coworkers^[^ ^61^ ^]^ used the self‐assembly approach to synthesize a nanosystem, which has high photothermal conversion efficiency and efficient packaging and controlled release capacity. The nanosystem loaded with DOX can be used as a combined photothermal/chemotherapy reagent to treat orthotopic xenotransplantation cancer. When carrying small molecule immunostimulants, this nanosystem shows a good therapeutic effect on mouse breast cancer model. In particular, the combined therapy not only cures the primary tumor, but also activates the antitumor immune activity of the whole body.

**Figure 2:**
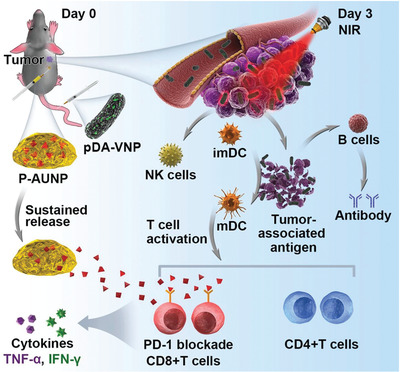
Triple combination of biotherapy, PTT, and sustained PD‐1 blockage therapy by pDA‐VNP to trigger robust antitumor immune response. Reproduced with permission.^[64]^ Copyright 2020, WILEY‐VCH Verlag GmbH & Co.

Since PTT can induce antitumor immunity of the body, rational use and appropriate enhancement of body's antitumor immune process can be effective for tumor treatment.^[^ ^59^, ^62^, ^63^ ^]^ In this case, Sun and coworkers^[^ ^64^ ^]^ encapsulated a photothermal material, polydopamine (pDA), on the surface of attenuated Salmonella VNP20009 (pDA‐VNP) through the oxidative self‐polymerization in alkaline solution (**Figure** **2**). Many kinds of immune cells participate in the therapy process. Bacteria‐carried photothermal materials can achieve efficient targeting to deep tumor hypoxic area and enhance the cancer cell killing effect in situ. Photothermal treatment kills tumors by high temperature in situ, producing tumor cell lysates that can be used as “self‐antigens.” On one hand, the self‐antigens can activate body's immune response. On the other hand, it can be employed as the nutrient, which is conducive to continue the reproduction of bacteria in the tumor site. In order to better apply this treatment strategy to large tumors that are difficult to overcome, they loaded PD‐1 immune checkpoint inhibitor into phospholipid phase change gel (P‐AUNP). A drug depot can be formed through single subcutaneous injection, and gradually released PD‐1 antagonist can last over 42 days, continuously changing the tumor immunosuppressive microenvironment, restoring the ability of T cells to kill the tumor, and thereby enhancing the antitumor effect of the nanosystem. This triple‐combined treatment strategy of bacteria, heat and immunity provides a fresh idea for tumor immunotherapy.

Combining PTT and PDT is also an important strategy, because many photosensitizing materials have both photothermal conversion ability and singlet oxygen generation capacity. For example, ICG is a clinical NIR fluorescent dye. It has not only strong photodynamic performance, but also a certain photothermal property. Therefore, it can be used for PDT and auxiliary PTT of tumor.^[^ ^65^, ^66^, ^67^ ^]^ However, ICG is usually unstable in the liquid environment, and could be easily cleared in the blood circulation, resulting in insufficient bioavailability and limited application in the tumor treatment. To address this issue, Kang and coworkers^[^ ^67^ ^]^ used cationic polymer polyethyleneimine to electrostatically adsorb ICG on the surface of magnetic Prussian blue nanoparticles to build a composite nanoplatform for the treatment of solid tumors. They found that ICG loaded nanocarrier can form stable aggregates, which can significantly increase their own circulation time in the blood. By establishing a solid tumor model in nude mice, highly efficient inhibition of tumors by light‐induced PTT/PDT was verified, showing promising clinical application prospects. Dong and coworkers^[^ ^68^ ^]^ then connected a monoamino‐substituted porphyrin photosensitizer to a covalent‐organic framework (COF), and used the inherent pores of the COF to load photothermal naphthalocyanine in order to increase the light stability. This synthetic strategy realizes the unification of photodynamic and photothermal treatment on a single platform. The experimental results show that the photothermal treatment could improve PDT by destroying lysosomes and mitochondria for enhanced therapeutic effect. Thus, the combined methods could achieve better tumor treatment outcome.

While combination therapy is an effective approach in cancer treatment, it is not simply a one‐plus‐one treatment method. We should exert the synergistic effect to achieve the therapeutic outcome of “one‐plus‐one greater than two,” rather than the simple combination of two therapeutic methods. For example, PTT combined with immunotherapy would need deeper understanding of antigen release after photothermal death of cancer cells, so that we will be able to use suitable immunotherapy drugs to gain the maximum efficacy.

#### 2.2.2 Bioresponsive Nanoagents

The tumor microenvironment is extremely complex, and many factors such as acidity, alkalinity, glutathione content, and oxygen content can affect the efficiency of PTT. Therefore, various photothermal materials based on the tumor microenvironment and other bioenvironments have constantly been developed.

Among these systems, polyoxometalate (POM) clusters, especially molybdenum (Mo)‐based POM, showed a great potential as photothermal agents for PTT of cancer. Shi et al.^[^ ^69^ ^]^ reported that Fe‐POM can be used not only as a PTT agent to produce the heat effect for killing cancer cells under NIR laser (1060 nm) irradiation, but also as a chemodynamic therapy (CDT) agent to convert H_2_O_2_ with low endogenous activity into harmful ·OH. It is worth noting that the increase in the body temperature induced by PTT can further enhance the CDT effect, achieving synergistic PTT/CDT. In another report, Yi and coworkers^[^ ^70^ ^]^ modified the structures of cyanine dyes through molecular design, and synthesized a pH‐responsive photothermal agent (pH‐PTT) that can sensitively respond to pH. The authors loaded bovine serum albumin (BSA) into this pH‐PTT to afford the pH‐responsive Golgi‐targeted PTT system. In a weakly acidic environment (pH = 5.0–6.5), this conjugated system showed strong absorption in the NIR region, which can effectively convert light energy into heat energy. Because the microenvironment of the Golgi is weakly acidic, and the Golgi in tumor cells is more diffused and hypertrophic than that in normal cells, this conjugated system can be more internalized by the tumor Golgi and triggered by the weak acid environment to convert NIR light into heat energy, thus realizing the “turn on” effect of photothermal property. Therefore, the combined therapy and enhanced PTT by bioresponsive nanoagents can both improve the efficiency of PTT, accelerating the PTT strategy toward clinical translations.

## 3 Solutions for the Defects in Photodynamic Therapy

### 3.1 Solutions for Enhancing Reactive Oxygen Species

As reported,^[^ ^71^ ^]^ the lifetime of ^1^O_2_ in aqueous solution is about 0–3 µs, and the diffusion distance is about 0–20 nm, which mean that the ROS generated by NIR light could be consumed in the cell membrane or cytoplasm, thus weakening its tumor killing ability.

#### 3.1.1 Application of Mitochondria Targeting in Solving Short Lifetime and Diffusion Distance of Reactive Oxygen Species

Mitochondria, the energy factory of cells, are mainly responsible for the metabolism of cells. It is also extremely sensitive to redox reactions, especially in tumor cells. Therefore, targeting mitochondria and interfering with mitochondrial function to achieve tumor killing have been proposed by researchers.

**Figure 3:**
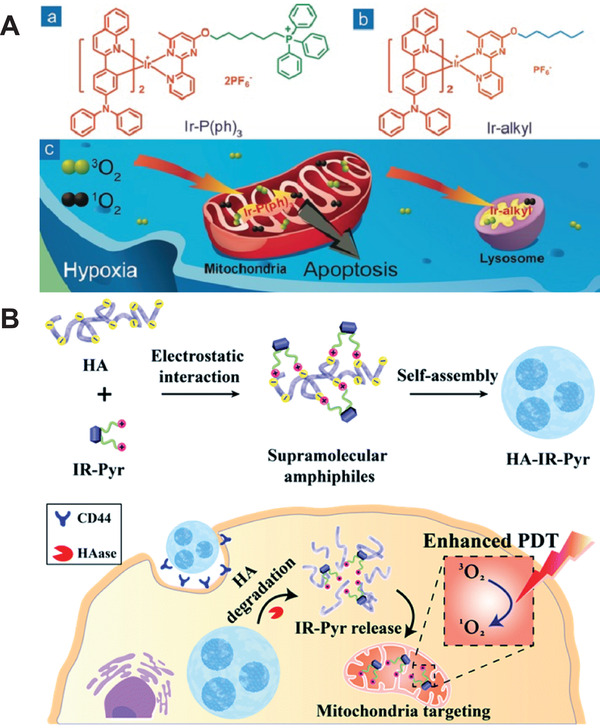
A) Chemical structures of the two iridium(III) complexes and their ability to target mitochondria and lysosomes for PDT. Reproduced with permission.^[72]^ Copyright 2016, WILEY‐VCH Verlag GmbH & Co. B) Preparation of IR‐Pyr‐encapsulated nanosystem for mitochondria‐targeted PDT. Reproduced with permission.^[75]^ Copyright 2017, Royal Society of Chemistry.

The development and use of mitochondrial targeting agents have also made a great progress in recent years. For example, Huang and coworkers^[^ ^72^ ^]^ reported two kinds of photosensitizers based on iridium(III) complexes (Ir‐P(ph)_3_ and Ir‐alkyl) for targeting mitochondria and lysosomes, respectively (**Figure** **3**A). They compared their photodynamic therapeutic effects under hypoxic conditions. Both photosensitizers exhibit long phosphorescent lifetime and sensitivity to oxygen. The mitochondria‐targeted complex has an inhibitory effect on mitochondrial aerobic respiration, showing high phototoxicity and photodynamic therapeutic effect in hypoxic environment. Thus, mitochondria‐targeted photosensitizers can be used to treat hypoxic tumor by PDT. He and coworkers^[^ ^73^ ^]^ designed and synthesized two iridium complexes with *β*‐carboline alkaloid ligands. Both complexes showed high anticancer activity. The cytotoxicity of these two compounds on lung cancer (A549) cells increased significantly under light (425 nm) exposure, because they highly localized in mitochondria. Cell‐based analysis showed that one of the iridium complexes could result in increased levels of intracellular ROS, mitochondrial DNA damage, increased lipid peroxidation levels, and inhibition of proteasome activity. Zhang and coworkers^[^ ^74^ ^]^ prepared chimeric peptide self‐delivery nanoparticles for mitochondrial and plasma membrane dual‐targeted PDT of tumor. In the absence of other carriers, the nanoparticles had high drug loading capacity and good ability to generate ROS. In addition, the dual targeting feature promotes effective subcellular localization of the photosensitizer for generating ROS in situ.

Some existing targeting agents could be improved to achieve better targeting effects, for example, an indocyanine dye derivative IR‐780 was improved by Ryu and coworkers^[^ ^75^ ^]^ IR‐780 is widely known for its PDT use, since it shows good absorption in the NIR region and mitochondrial targeting ability. However, poor water solubility, dark toxicity and photobleaching are its major drawbacks. The authors developed a water‐soluble indocyanine derivative (IR‐Pyr) having better light stability than IR‐780 (Figure 3B). In addition, the electrostatic interaction between positively charged IR‐Pyr and negatively charged hyaluronic acid was used to build micelles that were selective to cancer cells for high PDT efficacy. Hartman and coworkers^[^ ^76^ ^]^ used hyaluronic acid grafted phthalocyanine conjugate for tumor and mitochondrial targeted PDT. Compared with normal cells, the conjugate exhibits specific uptake in the mitochondria of breast cancer cells, giving light‐enhanced cytotoxicity. Thus, developing various types of new mitochondrial targeting agents and improving existing systems to enhance their mitochondrial targeting function would bring enhanced PDT efficacy.

Mitochondrial targeting‐based PDT not only resolves the drawbacks of short lifetime and diffusion distance of ROS, but also restricts ROS to specific organelles that are vulnerable to the ROS damage, so as to achieve the maximum cancer cell killing function. If the nanoparticle systems also have accurate tumor cell recognition ability, this method would minimize the damage to normal cells, showing a promising clinical application potential.

#### 3.1.2 Uniform Dispersion of Photosensitizers to Prevent Their Quenching

Porphyrin and phthalocyanine photosensitizers have the advantages of good biocompatibility and strong singlet oxygen generation ability, making them possible for clinical PDT. But, such photosensitizers are prone to have *π*–*π* stacking‐based aggregation with quenched performance. Therefore, dispersing the photosensitizers uniformly at molecular level to prevent their aggregation is an effective way to improve their photodynamic effect. Many functional materials such as mesoporous nanoenzymes^[^ ^77^ ^]^ and metal‐organic scaffolds can be used to prevent the aggregation effect.

For instance, Wei and coworkers^[^ ^78^ ^]^ reported a multifunctional delivery vehicle. The carrier consists of photosensitizer (zinc phthalocyanine) and targeting agent (folic acid) in polyvinylpyrrolidone micelles. The encapsulated folic acid achieves targeted cancer therapy due to the overexpression of folic acid receptor in cancer cells. They confirmed that zinc phthalocyanine was contained in the polymer micelles in a monomer state, thereby improving the efficiency of singlet oxygen generation. In vitro and in vivo studies showed the dispersed photosensitizer in the vehicle can improve singlet oxygen production capacity. Other materials such as COFs can perform similar functions. COFs are a type of porous materials with low density, high specific surface area, and precisely adjustable pore size, showing a great potential in the field of biomedical research. The high specific surface area offers a sufficient interface for the charge separation of the loaded photosensitizers. The internal *π* system provides a strong conductive path for the photosensitizers. The high volume promotes the diffusion of ROS or photons. Therefore, COFs are promising systems for photocatalysis and phototherapy applications. Pang and coworkers^[^ ^79^ ^]^ used a COF as the template to successfully synthesize COF–Ag_2_Se nanoparticles under mild conditions by the cation exchange method. The COF used in the study was not only a template for controlling the size of Ag_2_Se, but also served for PDT. Uniformly distributed photosensitizers present an advantage in PDT, and thus, the tumor killing ability was greater than conventional photosensitizers.

Therefore, mitochondrial targeting and preventing the quenching of photosensitizers are the main solutions to solve the problem of short singlet oxygen lifetime and short diffusion distance of ROS in PDT. Good dispersion of photosensitizers is a useful way to solve their photophysical quenching issue, which can maximize the application of photosensitizers in the cancer treatment.

#### 3.1.3 Photodynamic Therapy Assisted by Electroporation to Improve Photosensitizer/Drug Delivery

The effect of PDT depends on the effective transport of photosensitizers across the membrane and the intracellular accumulation of drugs. For some situations, the accumulation of photosensitizers and drugs is insufficient in tumor tissues, which may limit the availability of effective doses. Electric impulse could give action on cells through high‐speed and short‐term electrical stimulation, thereby opening transient non‐selective hydrophilic nanopores, serving as another way to cross lipid membranes. Under the action of electrical pulses, the cell membrane would be penetrated, resulting in increased permeability by various foreign molecules. To confirm the effect of electroporation with PDT,^[^ ^80^ ^]^ after electroporation, mouse liver cancer MH22A cells were exposed to light in vitro in the presence of a photosensitizer (chlorin e6 (Ce6) or aluminum phthalocyanine aluminum tetrasulfonate). The accumulation of the photosensitizer was recorded by fluorescence microscope, and the results indicated that electroporation can improve the uptake of Ce6 and aluminum phthalocyanine tetrasulfonate into MH22A cells, which significantly reduced the viability of the treated cells even at low doses of the photosensitizer. Although the significant uptake of photosensitizers has been observed in vitro, how to make this application in vivo would still need more exploration.

### 3.2 Solutions for Hypoxia

**Figure 4:**
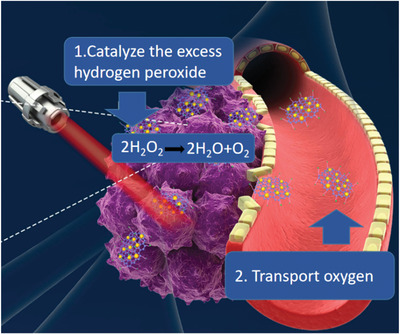
Two main research aspects to improve hypoxia: 1) catalyze the excess hydrogen peroxide inside the tumor to generate oxygen in situ for oxygen supply and 2) transport oxygen from other parts to the tumor.

Hypoxic microenvironment is a common feature of solid tumors. Hypoxic tumor microenvironment is caused by increased oxygen consumption and decreased oxygen transport. A series of physiological responses caused by hypoxia is mainly driven by hypoxia‐inducible factor (HIF). During hypoxia, the level of HIF1 protein increases, and the HIF‐mediated pathway affects metabolic adaptation through mammalian target of rapamycin (mTOR) signaling, erythropoiesis, angiogenesis, cell growth, vascular tone, and differentiation. Hypoxia is generally considered as a factor that promotes the tumor development, such as hypoxia‐induced tumor chemotherapy resistance^[^ ^81^, ^82^ ^]^ and epithelial‐mesenchymal transformation.^[^ ^83^, ^84^, ^85^ ^]^ Hypoxia is also a factor that must be considered in PDT, because oxygen is the third necessary factor in addition to photosensitizers and NIR light. Only the presence of oxygen can produce singlet oxygen, thereby playing the PDT role of tumor killing effect. Therefore, improving the tumor hypoxic microenvironment is an important part of increasing the efficacy of PDT. The current research focuses on two aspects to improve hypoxia (**Figure** **4**). One is to catalyze the excess hydrogen peroxide inside the tumor to generate oxygen in situ for oxygen supply, and the other is transporting oxygen from other parts to the tumor region.

#### 3.2.1 Catalyzing Hydrogen Peroxide Inside Tumor to Generate Oxygen In Situ

Numerous studies have shown^[^ ^86^, ^87^, ^88^, ^89^ ^]^ that there is a certain amount of hydrogen peroxide inside the tumor tissue. This endogenous H_2_O_2_ in the tumor can be catalytically decomposed to generate oxygen (O_2_) in situ, thereby solving the problem of hypoxia and improving the efficacy of PDT. Accordingly, H_2_O_2_ has a great potential as an internal responsive stimulus. The development of nanomaterials based on H_2_O_2_ response can improve the treatment efficiency of cancer and promote the clinical development of PDT. H_2_O_2_‐responsive materials include inorganic materials and organic materials. Inorganic materials normally include manganese‐based materials, platinum‐based materials, iron‐based materials and copper‐based materials. Due to their high reactivity to H_2_O_2_, they can serve as H_2_O_2_‐driven materials and oxygenating agents, thus increasing the oxygen concentration and ultimately promoting the efficacy of PDT. Organic materials include those systems with aryl oxalate bond (—OCOCOO—), sulfur bond (—S—), selenium bond (—Se—), tellurium bond (—Te—), and boric acid bond that are responsive to H_2_O_2_.

**Figure 5:**
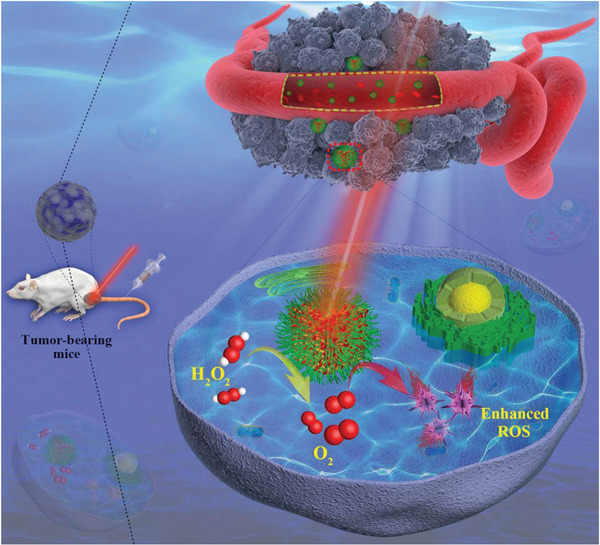
A mesoporous nanoenzyme used as the scaffold for uniformly dispersing the loaded photosensitizers to achieve enhanced PDT of tumor. Reproduced with permission.^[77]^ Copyright 2019, WILEY‐VCH Verlag GmbH & Co.

Zhao and coworkers^[^ ^77^ ^]^ proposed a mesoporous nanozyme (NE) derived from a MOF, which had H_2_O_2_ catalytic ability to generate endogenous O_2_ in situ and can enhance the efficacy of PDT under the guidance of bioimaging (**Figure** **5**). This mesoporous nanozyme was loaded with Ce6, a commonly used photosensitizer in PDT, with a high loading capacity. Once this nanozyme catalyzes the endogenous hydrogen peroxide to generate O_2_, the hypoxic tumor microenvironment is relieved. Therefore, this Ce6‐loaded nanozyme can be used as H_2_O_2_ catalyst to increase the local O_2_ concentration, thereby significantly enhancing the efficacy of antitumor PDT in vitro and in vivo. In another example, Liu and coworkers^[^ ^90^ ^]^ developed 1D palladium nanosheets with PDT ability, H_2_O_2_ catalytic capability, and biodegradability, which can realize photothermal and photodynamic collaborative treatment under the excitation of NIR light. Due to the porous nature of palladium nanosheets, they can efficiently catalyze the decomposition of hydrogen peroxide to generate a large amount of oxygen, overcoming the tumor hypoxia and enhancing the PDT effect. Peng and coworkers^[^ ^91^ ^]^ developed a unique liposome‐encapsulated catalase, which can respond to an NIR photosensitizer. When loaded with DOX, the hybrid liposome can catalyze highly expressed H_2_O_2_ to increase tumor oxygenation, thereby enhancing the combinational efficacy of chemotherapy and PDT. In addition, enhanced tumor oxygenation not only promotes the production of singlet oxygen (^1^O_2_), but also reverses the immunosuppressed tumor microenvironment by regulating immune cytokines, which are beneficial to the antitumor immunity to further induce the tumor death.

This type of treatment that catalyzes H_2_O_2_ to generate oxygen inside tumor tissue to improve the hypoxic environment often integrates PDT with other treatment options to achieve enhanced therapeutic outcome. It is believed that this combination strategy would realize the strongest tumor inhibition effect.

#### 3.2.2 Transporting Oxygen from Other Sites to Tumor

In addition to generating oxygen in situ to improve the hypoxic environment, researchers also explored other ways, such as transporting oxygen through biological materials or cells in the body to the tumor site, thereby improving the hypoxic environment of the tumor.

Red blood cells are the most blood cells in the blood. They are responsible for the transportation of nutrients and oxygen in the body to maintain the normal operation of our body. Is it possible to use the function of red blood cells to carry oxygen to solve the hypoxic problem of tumor, improving the therapeutic effect of tumors? To answer this question, Zhang and coworkers^[^ ^92^ ^]^ prepared aggressive artificial red blood cells to overcome the resistance of tumor tissue hypoxia. Hemoglobin is easily oxidized during the circulation, losing its oxygen‐carrying capacity and producing toxic substances such as high‐valent hemoglobin. In order to resist oxidative damage to hemoglobin, red blood cells have a natural antioxidant system including special enzymes, superoxide dismutase and catalase. Inspired by the biological characteristics of red blood cells, this study designed artificial red blood cells to achieve self‐oxygenated tumor PDT. Specifically, artificial red blood cells were prepared by encapsulating hemoglobin, pDA and photosensitizer in recombinant erythrocyte membrane vesicles. Among them, hemoglobin carries oxygen as an oxygen carrier, pDA mimics the function of antioxidant enzymes in natural red blood cells to prevent hemoglobin oxidation, and at the same time, aromatic rings of pDA provide adsorption sites for photosensitizers or chemotherapeutic drugs. The outer layer of red blood cell membrane vesicles improves the biocompatibility and achieves long circulation in the body. Experiments showed that the artificial red blood cells can be enriched at the tumor site, improving the hypoxic condition to achieve better PDT effect for killing the tumor.

In another case, Feng and coworkers^[^ ^93^ ^]^ took a different approach to prepare an oxygen‐reducing nanoplatform, which could inhibit the aerobic respiration of cells and reduce the oxygen consumption of tumor cells to achieve the purpose of hypoxia relief, thereby enhancing the effect of PDT. In this study, a biodegradable polymer poly(d,l‐lactide‐*co*‐glycolide) (PLGA) was used to form a nanovesicle carrier. A hydrophobic photosensitizer was encapsulated in the hydrophobic layer of PLGA vesicles, and a hydrophilic nitric oxide (NO) donor was encapsulated in the hydrophilic cavity of the vesicles. When the vesicles reach the tumor site, the released NO donor reacts with overexpressed glutathione to produce NO. The resulted NO can inhibit cellular respiration and reduce cellular oxygen consumption, effectively alleviating the hypoxic symptom at the tumor site. This study presents an oxygen generation nanodevice to enhance PDT by inhibiting the respiration of tumor cells, providing an interesting idea for the treatment of hypoxic tumor. Cai and coworkers^[^ ^94^ ^]^ used a complex of ICG photosensitizer/hemoglobin oxygen carrier to cover the phospholipid layer in order to mimic the red blood cell membrane. The constructed artificial red blood cells have oxygen carrying and oxygen releasing functions. When entered into the tumor, the released oxygen would relieve hypoxic microenvironment of tumor and increase the antitumor performance of PDT.

A major issue of PDT for antitumor application is the lack of oxygen at the tumor site, which negatively affects the production of toxic singlet oxygen. Therefore, improving the tumor hypoxia microenvironment can boost the PDT effect. In addition to catalyzing intracellular hydrogen peroxide at the tumor site, we can also transport oxygen to the tumor using rationally designed nanocarriers, and then PDT would perform efficiently.

### 3.3 Solutions for Poor Penetration of Excitation Light Source

No matter how efficient the phototherapy is, it is still difficult for PTT and PDT to solve the issue of insufficient penetration of NIR light. NIR light at 808 nm is widely used in PDT, but its penetration depth is only 1–2 mm. Clinically, it is mainly used for the treatment of superficial tumor tissues such as skin cancer. For deep tumors, how to enable the excitation power to reach its location is the key problem to be addressed.

#### 3.3.1 Sonodynamic Therapy as an Alternative Approach

While hematoporphyrin for PDT alone or ultrasound alone has only a slight inhibitory effect on the growth of transplanted tumor in mice, synergistic inhibition effect of ultrasound combined with hematoporphyrin is significant. This combination therapy is named sonodynamic therapy (SDT). When providing ultrasound to the sound‐sensitive substances (such as porphyrins) localized in tumor cells, the absorption capacity undergoes an electronic transition from low energy to high energy states. After returning to the low energy state, a large amount of energy is released to stimulate hematoporphyrin to produce trivalent hematoporphyrin. Because trivalent hematoporphyrin is extremely unstable, it quickly splits into monovalent hematoporphyrin, releasing singlet oxygen radical with strong oxidizability to effectively cause the tumor cell death. The thrombus formed by the inflammatory reaction of tumor blood vessels could also result in ischemic necrosis of tumor tissue. SDT, an alternative treatment for PDT, is gradually accepted by the researchers, showing a tremendous application potential in the field.^[^ ^95^ ^]^

**Figure 6:**
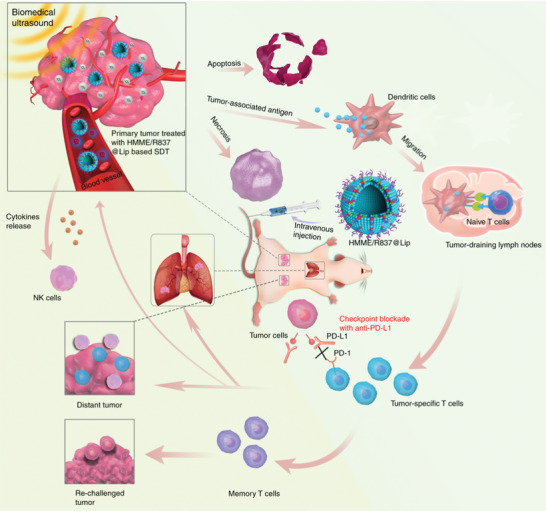
Schematic illustration of the combination therapy based on sonosensitizer‐associated SDT and anti‐PD‐L1 checkpoint blockade immunotherapy for enhanced tumor treatment. Reproduced with permission.^[96]^ Copyright 2019, Springer Nature Limited.

Similar to PDT, SDT utilizes powerful penetration power of ultrasound to penetrate into deep tissue, activates the sonosensitizers to produce ROS, and then kills cancer cells to achieve the therapeutic purpose. Chen and coworkers^[^ ^96^ ^]^ reported a combination therapy based on sonosensitizer‐associated SDT and anti‐PD‐L1 checkpoint blockade immunotherapy for enhanced tumor treatment (**Figure** **6**). In this system, liposomes act as the carrier to encapsulate the sonosensitizers. It was proven that the combination of SDT and anti‐PD‐L1 treatment can induce the antitumor response, which not only prevents primary tumor progression, but also inhibits lung metastasis. In addition, the combined treatment provides long‐term immune memory function, which can stop the tumor from the recurrence after eliminating the initial tumor. Therefore, the SDT approach could solve the issue of insufficient penetration depth of NIR light in PDT.

Since we mainly discuss the solutions to the problems existing in phototherapy, we would not introduce SDT in more detail. However, because many photosensitizers have been found to have acoustic sensitivity, integrating SDT with phototherapy should be a promising solution. High tissue penetration ability of ultrasound has indeed brought new ideas for the treatment of deep solid tumors.

#### 3.3.2 Self‐Activated Photodynamic Therapy

The interaction between radionuclides and nanomaterials can produce Cerenkov radiation (CR) for CR‐induced PDT (CRIT) without external light excitation. Radiation refers to the transmission of energy in the form of waves or subatomic particles. Some radiation forms have already been used in the clinic, such as the radionuclide diagnosis in positron emission tomography. CR is a short‐wavelength electromagnetic radiation characterized by blue glow. Kotagiri et al.^[^ ^97^ ^]^ used CR emitted by radionuclides to activate titanium dioxide nanoparticles in order to generate ROS for the tumor treatment. The main method is to use transferrin‐covered TiO_2_ and clinically applied radionuclides to colocalize in mouse tumor tissues for achieving the CRIT effect. Ni et al.^[^ ^98^ ^]^ employed ^89^Zr radionuclide and a porphyrin molecule (TCPP) to modify the surface of magnetic nanoparticles (MNPs), affording ^89^Zr‐MNPs/TCPP as a therapeutic agent for CR‐induced magnetic targeting PDT. The system exhibits high tumor accumulation under an external magnetic field. Through the guidance of multi‐modal imaging including fluorescence, CR luminescence, and CR resonance energy transfer, this system exerts a good PDT effect on tumor. Other CR sources include ^68^Ga‐labeled bovine serum albumin (^68^Ga‐BSA)^[^ ^99^ ^]^ and ^89^Zr‐labeled deferoxamine‐porphyrin nanocomplex.^[^ ^100^ ^]^

The radionuclide‐derived CR approach can be used as a built‐in light source for PDT, opening up new horizons for biomedical applications. However, the tumor targeting efficiency of existing radionuclides is relatively low. It is a challenge to accurately and efficiently deliver CR energy to the tumor site. Thus, this kind of PDT still requires new strategies to improve and amplify the treatment efficiency when used in the clinic.

#### 3.3.3 Deep‐Tissue Photodynamic Therapy by X‐Ray Radiation

X‐ray radiation has stronger tissue penetrating ability than NIR light and can efficiently activate PDT from almost any part of the body.^[^ ^101^ ^]^ Chen and coworkers employed X‐ray to activate PDT in order to kill tumor cells in vitro.^[^ ^102^ ^]^ Then, more studies of X‐ray‐induced PDT have been reported. Shrestha et al.^[^ ^103^ ^]^ found that copper‐cysteamine nanoparticles could be activated directly by X‐ray to produce singlet oxygen. This work confirmed the effectiveness of copper‐cysteamine nanoparticles as photosensitizers when activated by X‐ray radiation, and showed promising aspects for deep tumor PDT. Yang et al.^[^ ^104^ ^]^ reported that carbon‐doped TiO_2_ could be activated by X‐ray for the enhanced PDT in the tumor treatment. The carbon‐doped TiO_2_ acts as a photosensitizer in the process of low‐dose X‐ray‐induced PDT, which overcomes the limitation of penetration depth and radiation dose. Yu et al.^[^ ^105^ ^]^ developed a multifunctional nanosystem using merocyanine 540‐modified Gd_2_(WO_4_)_3_:Tb. The nanosystem had the ability to realize X‐ray‐induced photodynamic/radiation cotherapy of tumor. Their experiments proved that, compared with single radiotherapy, this synergistic therapy approach had higher tumor inhibition efficiency with even low dose X‐ray radiation.

Although some low‐dose X‐ray‐induced PDT methods have been proposed, inducing PDT normally requires at least 5Gy X‐ray irradiation to achieve the expected therapeutic outcome, which is equivalent to or even higher than the dose used in clinical radiotherapy.^[^ ^106^, ^107^ ^]^ In addition, side effects of X‐ray should also be taken into consideration in further research. In view of the good penetration feature and radiotherapy ability of X‐ray, X‐ray‐induced PDT and combined therapy could play an important role in the treatment of deep tumors.

#### 3.3.4 Specific Generation of Singlet Oxygen

Whatever we do to enhance the penetration effect of irradiation source, several defects such as oxygen dependence, limited penetration depth of external light, and low treatment efficiency still exist. In order to arrive the key inhibitory effect, a variety of nanoscale O_2_ release and glutathione consumption based photodynamic agents have been developed to relieve tumor hypoxia or reduce the glutathione concentration. Recently, Jiang and coworkers^[^ ^108^ ^]^ reported 2D MOF nanosheets (Cu‐TCPP) for selectively producing ^1^O_2_ in tumor microenvironment. Intracellular H_2_O_2_ peroxidizes the MOF ligand to generate peroxyl radicals, and the nanosheets can also consume glutathione. Therefore, Cu‐TCPP nanosheets can efficiently and selectively inhibit the tumor growth, indicating an effective way to overcome the limitations of PDT.

Nanosystems such as Cu‐TCPP nanosheets can effectively produce ^1^O_2_, and at the same time, deplete glutathione, thereby inducing the tumor apoptosis without serious side effects. Obviously, this method efficiently avoids the dependence of PDT on oxygen and external light irradiation, which provides meaningful inspiration and guidance for further development of cancer treatment strategies.

### 3.4 Solutions for Insufficient Reactive Oxygen Species of Monotherapy

Similar to PTT, sole PDT is often difficult to achieve full tumor killing function due to limited singlet oxygen generation capacity and limited action distance. Therefore, the combination of PDT with other treatment methods has been a research hotspot. Since PDT has shown to induce immunogenic death of tumor cells,^[^ ^22^, ^109^, ^110^, ^111^, ^112^, ^113^, ^114^ ^]^ the combination of PDT with immunotherapy is an ideal choice for research.

Lin and coworkers^[^ ^115^ ^]^ loaded anticancer drug oxaliplatin and photosensitizer into core‐shell nanoparticles in order to improve the efficacy of immune checkpoint therapy for colon cancer. On one hand, the anticancer drug oxaliplatin can cause immunogenic cell death. On the other hand, the photosensitizer can generate reactive oxygen radicals under light, which not only kill tumor by apoptosis or necrosis, but also stimulate the immune system, produce acute inflammation, and allow leukocytes to infiltrate into the tumor, thereby improving the presence of tumor‐associated antigens to T cells. When this nanomedicine is combined with PD‐L1 checkpoint blockade therapy, it eliminates the tumor in situ, and also inhibits metastatic tumor by activating the immune system. After that, the authors^[^ ^116^ ^]^ designed nanoscale MOFs loaded with checkpoint inhibitors to treat colon cancer. The chlorin species in MOFs can serve as a photosensitizer to generate reactive oxygen radicals under light, resulting in immunogenic cell death and in turn activating the immune system to promote the antigen express. In addition, the loading of checkpoint inhibitors can change the immunosuppressive tumor environment. Thus, the synergistic effect not only eliminates the tumor in situ, but also creates an immunogenic microenvironment to prevent the tumor metastasis.

In order to further confirm that PDT can increase the effectiveness of immune checkpoint blockage therapy, in the follow‐up studies,^[^ ^117^ ^]^ they selected breast cancer that was insensitive to the immunologic drugs used. In this experiment, they found that PD‐L1 antibody‐mediated immune checkpoint blockage therapy produces a curative effect on breast cancer, and combining PDT with PD‐L1 antibody effectively eliminates the tumor in situ and prevents the metastasis of the tumor. These studies show that PDT plays two roles in sensitizing the tumor environment. On one hand, PDT induces immunogenic cell death and releases tumor‐associated antigens to T cells to produce tumor‐specific therapeutic effects. On the other hand, PDT can create an inflammatory environment, which enhances the ability of T cells and other immune cells to penetrate the tumor for the treatment.

**Figure 7:**
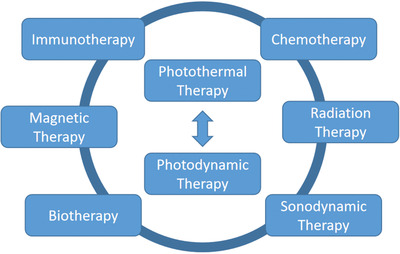
Combination of different therapy methods with PTT and PDT. Drawn by Adobe Illustrator.

In addition to the combination with immunotherapy, the combined use of PDT with other treatment approaches has also been developed to show tremendous antitumor effect (**Figure** **7**). For instance, the combination of PDT with magnetic therapy is a relatively new field. Dong and coworkers^[^ ^118^ ^]^ recently developed a Janus‐type nanosystem consisting of ferromagnetic tetroxide ball and mesoporous silica rod. The asymmetric structure has the feature that functional components do not interfere with each other. The magnetic ball has the superparamagnetism and can be used for magnetocaloric therapy with magnetic targeting ability. The mesoporous silica rod can efficiently encapsulate the Ce6 photosensitizer and degrade in the tumor microenvironment. At the same time, the tumor cell membrane is coated on the surface of the nanosystem, which prolongs its blood circulation time and improves its targeting effect to the tumor tissue. Therefore, an efficient synergistic antitumor effect and a tumor‐specific immune response were achieved. When combined with the immune checkpoint blocker, cytotoxic T cell antigen‐4 antibody, stronger systemic antitumor immune response was produced. In addition to the combination between PDT and chemotherapy,^[^ ^119^, ^120^, ^121^ ^]^ there are other combination strategies, which would not be detailed. In short, PDT combined with other treatment approaches can maximize their advantages and achieve a complete tumor suppression effect. In addition, what we should pay more attention to is the synergistic effect of combination therapy. As discussed above, studies have already confirmed the immune activation ability after PDT. Making full use of the features would further promote the therapeutic efficacy for the cancer inhibition.

## 4 Clinical Prospects and Conclusions

This article summarizes recent research development of PDT and PTT by using well designed nanosystems. As emerging adjunct treatment methods, PTT and PDT show a great potential in clinical applications toward cancer treatment, as long as they can overcome their own shortcomings. In fact, some clinical trials using PDT approach have been performed for treating certain types of cancer. A systematic review^[^ ^122^ ^]^ searched and selected 21 clinical trials published during 1991–2017 that were in line with randomized controlled trials related to PDT for human cervical intraepithelial neoplasia (CIN) and human papilloma virus infection. In all, 64.2% patients in the PDT group achieved primary complete remission at the end of the three‐month follow‐up period, as confirmed by cytology and histology. 36.4% patients in the placebo group achieved complete remission. Thus, PDT significantly increased the complete remission rate of CIN in the overall test. This conclusion confirmed the clinical effect of PDT. On the other hand, we cannot ignore the side effect of PDT. Data showed that PDT resulted in a considerably higher rate of adverse events than the placebo group. The main adverse reactions were local discomfort, burning sensation, and mild to moderate vaginal discharge. Compared with the excellent treatment outcome, these adverse reactions are within the acceptable range of patients.

Other clinical trials of PDT include basal cell carcinoma, squamous cell carcinoma in situ,^[^ ^123^ ^]^ colorectal cancer,^[^ ^124^ ^]^ and high‐grade gliomas.^[^ ^125^ ^]^ Compared with the control group or the placebo group, all these clinical trials of PDT have achieved better antitumor effects. More importantly, researchers also observed the stimulation of antitumor immune response in the clinical trials,^[^ ^125^ ^]^ which has a positive effect on long‐term inhibition of tumors. PDT plays an important role in prolonging the survival time of patients and reducing the pain of other palliative treatments. Nevertheless, continued clinical studies should be conducted to provide more evidence‐based applications of PDT.

Based on this perspective, we discussed the main drawbacks that prevent PTT and PDT from further applications, as well as corresponding solutions that researchers have proposed. Major issues associated with phototherapy were first discussed, and corresponding solutions proposed by researchers were then highlighted. The phototherapy includes two major treatment processes, i.e., PTT and PDT. Both of them have the advantages of their highly efficient therapeutic effect and noninvasive treatment nature.

During the continuous investigations of phototherapy, some major issues have been identified. The thermal damage of photothermal treatment to normal tissues discussed in this article can be solved by improving the targeting ability of photothermal materials and low‐temperature photothermal treatment. While these two methods are both efficient, low‐temperature photothermal effect would still need more investigations, because the tumor killing mechanism is not clear enough. In addition, loading more drugs and proteins into therapeutic nanosystems, may affect the effectiveness of photosensitizers. Therefore, how to balance the killing efficiency and normal issue protection ability should be taken into consideration in future research. The issue of insufficient sole phototherapy treatment can be solved by integrating with other treatment options. Synergistic effect by these treatment approaches should be well explored, rather than a simple combination of different therapeutic approaches. Among all these therapeutic strategies, immunotherapy presents great advantages for its systemic regulation ability, which not only inhibits the tumor in situ, but also facilitates to reduce the rate of the recurrence and metastasis.

ROS could be easily quenched. The issues of extremely short lifetime and short diffusion distance can be solved by targeting mitochondria and uniformly dispersing the photosensitizers to prevent the quenching of the photosensitizers caused by *π*‐*π* stacking interactions. As the largest energy factory in cancer cells, mitochondria are very sensitive to oxygen and ROS. Targeting mitochondria can gain the maximum efficiency of the cancer therapy. The lack of sufficient oxygen in tumor can be solved by catalyzing excess hydrogen peroxide inside the tumor and transporting oxygen from other parts to the tumor site. In addition to the enhanced photothermal and photodynamic therapy, improving the tumor hypoxic microenvironment is also important means to inhibit the tumor growth and metastasis. Thus, developing novel nanosystems for efficient oxygen transportation and catalyzing hydrogen peroxide is a desired approach. The issue of poor penetration efficiency of the ROS excitation light source can be addressed by changing the excitation source, for example, using more penetrating ultrasound source and X‐ray radiation. In addition, self‐activated PDT by CR or other special means can also be used to gain deeper tissue penetration effect. The problem of insufficient ROS‐based single treatment can be solved by combining it with other treatment methods and making full use of tumor cell immune death after PDT.

This article highlights the existing solutions to some major problems associated with PTT and PDT. However, we understand that there are still other issues in phototherapy, and more possible solutions should be discovered to solve these issues. Thus, it is believed that most issues in phototherapy can be solved upon further studies, and phototherapy will play more and more important roles in clinical cancer treatment.

## Conflict of Interest

The authors declare no conflict of interest.
